# Whole Transcriptome Analysis Identifies TNS4 as a Key Effector of Cetuximab and a Regulator of the Oncogenic Activity of KRAS Mutant Colorectal Cancer Cell Lines

**DOI:** 10.3390/cells8080878

**Published:** 2019-08-12

**Authors:** Sujin Kim, Nayoung Kim, Keunsoo Kang, Wonkyung Kim, Jonghwa Won, Jeonghee Cho

**Affiliations:** 1 Department of Nanobiomedical Science, Dankook University, Cheonan 31116, Korea; 2 Department of Microbiology, Dankook University, Cheonan 31116, Korea; 3 Oncology Team, Mogam Institute for Biomedical Research (MIBR), Yongin 16924, Korea

**Keywords:** TNS4, KRAS, EGFR, cetuximab, selumetinib, colon cancer

## Abstract

The targeting of activated epidermal growth factor receptor (EGFR) with therapeutic anti-EGFR monoclonal antibodies (mAbs) such as cetuximab and panitumumab has been used as an effective strategy in the treatment of colorectal cancer (CRC). However, its clinical efficacy occurs only in a limited number of patients. Here, we performed whole-transcriptome analysis in xenograft mouse tumors induced by *KRAS*^G12D^ mutation-bearing LS174T CRC cells following treatment with either cetuximab or PBS. Through integrated analyses of differential gene expression with TCGA and CCLE public database, we identified TNS4, overexpressed in CRC patients and *KRAS* mutation-harboring CRC cell lines, significantly downregulated by cetuximab. While ablation of TNS4 expression via shRNA results in significant growth inhibition of LS174T, DLD1, WiDr, and DiFi CRC cell lines, conversely, its ectopic expression increases the oncogenic growth of these cells. Furthermore, TNS4 expression is transcriptionally regulated by MAP kinase signaling pathway. Consistent with this finding, selumetinib, a MEK1/2 inhibitor, suppressed oncogenic activity of CRC cells, and this effect is more profound in combination with cetuximab. Altogether, we propose that TNS4 plays a crucial role in CRC tumorigenesis, and that suppression of TNS4 would be an effective therapeutic strategy in treating a subset of cetuximab-refractory CRC patients including *KRAS* activating mutations.

## 1. Introduction

Colorectal cancer (CRC) is the third most common type of cancer worldwide and a five-year survival rate was reported among less than 10% of metastatic colorectal cancer (mCRC) patients [1,2]. Currently, FDA-approved cetuximab and panitumumab anti-EGFR monoclonal antibodies (mAb), in combination with either FOLFIRI or FOLFOX, are widely used as a standard of care for the majority of mCRC patients [3,4,5]. However, the clinical response of the cetuximab or panitumumab directed therapeutic option is achieved in only 10~20% of unselected patients [6,7].

The main established mode of antitumor action of cetuximab and panitumumab is to suppress the dysregulated mitogenic signaling pathways such as RAS/MEK/MAPK and PI3K/AKT by inhibiting aberrant EGFR activation via blocking the binding of its various ligands upon the receptor [8,9]. Therefore, it provides a rationale that the CRC patients harboring activating mutations in *KRAS* (~45%) or *NRAS* (~5%), which function as a strong inducer of these signaling cascades irrespective of EGFR, are refractory to both cetuximab and panitumumab directed therapy [10,11,12,13,14]. Besides to *RAS* mutations, it has been clinically proven that genomic alterations targeting *BRAF*, *PIK3CA,* or *ERBB2*, loss of *PTEN* and amplification of *EGFR* or *ERBB2* are now known to be negative predictors associated with the drug efficacy among mCRC patients [15,16,17,18,19]. Notably, given that no adequate inhibitor targeting mutant RAS is clinically available, there is a high demand for the development of effective therapeutic approaches to treat more than 50% of mCRC patients harboring activating *RAS* mutations [18,20,21].

Interestingly, recent studies showed that EGFR signaling inputs are still required for initiation and progression of KRAS^G12D^-driven lung tumor formation in humans and in mice [22,23]. Furthermore, these reports demonstrated compelling evidence that EGFR-targeted drugs effectively enhance the therapeutic benefit of MAP kinase inhibition using in-vivo models [22]. Thus, these results suggest that inhibition of EGFR signaling cascade is still beneficial to antitumor effects in KRAS mutant-driven tumorigenesis and EGFR-targeted drugs such as cetuximab and panitumumab can be used as crucial therapeutic options for treating a subset of cancer patients harboring *KRAS* activating mutations.

In this study, to investigate the pharmacological effects of cetuximab in KRAS mutant-bearing CRC, we systematically examined the global expression changes in cetuximab-treated xenograft mouse tumors generated with KRAS mutant-harboring LS174T colorectal cancer cells and identified that significantly downregulated TNS4 by cetuximab is closely associated with oncogenic potential of a subset of CRC cell lines harboring *KRAS* activating mutations.

## 2. Materials and Methods

### 2.1. Generation of Tumor Xenograft Mouse

Female athymic mice (Charles River, Japan) were maintained in a pathogen-free colony and acclimatized for a week before being used. All studies were done in accordance with Laboratory Animal Care guidelines of Mogam Institute for Biomedical Research and approved by Mogam Biotechnology Institute (Approval number GC13-101A). LS174T cells (5 × 10^6^ cells, American Type Culture Collection) in 200 mL of PBS were injected subcutaneously into the flanks of BALB/c-nu/nu mice of 6–8 weeks of age. Tumor size was measured two times a week using a Vernier caliper and tumor volumes were calculated according to the formula of (short diameter)^2^ × (long diameter)/2. When tumor volume reached around 500 mm^3^, mice were randomized into each group. After confirming that mean tumor volumes were not statistically different among groups, mice were administered with PBS or cetuximab (Erbitux, Merck, Darmstadt, Germany) (1 mg/mouse) intra-peritoneally. Tumor xenografts were harvested 24 h after each treatment, frozen by liquid nitrogen, and stored at −80 °C.

### 2.2. RNA-seq Analysis

Total RNA was taken from a cohort of 4 mice tumors for each group. The library was prepared using the TruSeq RNA sample preparation kit (Illumina, San Diego, CA, USA) and sequencing was performed with Illumina Hiseq2500. Low-quality portions of sequenced reads were trimmed using Trim galore (https://github.com/FelixKrueger/TrimGalore). To avoid potential contamination from nearby mouse cells, the human (hg38) and mouse (mm10) reference gene sequences were merged into one FASTA file and indexed together using HISAT2 [24] with default parameters. Then, the trimmed reads were aligned to the merged transcriptome using HISAT2. RSEM [25] was used to quantify the abundance of all human and mouse known genes, and the genes belonging to the mouse transcriptome were discarded. EBSeq [26] was used to identify differentially expressed genes (DEGs) between groups (four biological replicates for each group) with a false discovery rate (FDR)-adjusted *p*-value cutoff of 0.05.

### 2.3. Cancer Cell Line Encyclopedia (CCLE) Data Analysis

Gene-centric RMA-normalized mRNA expression data for 1168 cell lines, including 61 colon cancer cell lines, was downloaded from the CCLE database (https://portals.broadinstitute.org/ccle). Differences in expression of a given gene between colon cancer and the other cell lines were tested using the Mann-Whitney U test (*p*-value < 0.05).

### 2.4. The Cancer Genome Atlas (TCGA) Data Analysis

Expression levels of known genes in colorectal adenocarcinoma (COADREAD) and adjacent normal tissues were downloaded from the TCGA database (http://firebrowse.org/). Totals of 382 tumor and 51 normal samples (mRNASeq) were compared to our RNA-seq data. For all known genes, the fold change was calculated by dividing the mean of expression values in tumor by the mean of expression values in normal. Then, log2 of the fold change was used.

### 2.5. Gene Set Enrichment Analysis

Gene set enrichment analysis (GSEA) was performed after filtering out genes (adjusted *p*-value > 0.05) [27]. A total of 5367 genes were used for the GSEA analysis.

### 2.6. Cell Culture and Reagents

LS174T, DLD1, WiDr, and DiFi cells were purchased from the American Type Culture Collection (ATCC, Manassas, VA, USA). The cell lines were maintained in RPMI with 10% FBS. The overexpressed TNS4 in LS174T, DLD1, WiDr, and DiFi cell lines were established by lentiviral infections, pooled and maintained as previously described [28]. PLX302 TNS4 expression plasmid was obtained from The ORFeome Collaboration (Dana-Farber Cancer Institute). Plasmid sequence was verified via sanger sequencing. To prepare the lysates and extract the RNA, the cells were prepared serum-starved for 18 h before EGF stimulation or conducted drug treatment in 2% FBS for 24 h. Cetuximab was purchased from Merck. Afatinib (LC laboratories, Woburn, MA, USA) and selumetinib (Selleckchem, Houston, TX, USA) were dissolved in DMSO at 10 mM. Epidermal growth factor (EGF, Invitrogen, Waltham, MA, USA) stimulation was performed at 10 ng/mL for 12 h.

### 2.7. Anchorage-Independent Growth Assay

Soft agar assays were performed in triplicate, as previously described [29]. 2–3 weeks later, the colonies were confirmed using dissection microscope and then taken images by eXcope program. The number of colonies was quantified using Image J software (version 1.5, NIH, Bethesda, MD, USA). The data were expressed as relative ratio in the graph after normalization of the comparative group as a control group. Each analysis was repeated at least twice with comparable results.

### 2.8. Cell Growth Inhibition Assay

The LS174T (8 × 10^3^), DLD1 (8 × 10^3^), WiDr (8 × 10^3^) or DiFi (8 × 10^3^) cells were seeded in 160-180 μL media in 96-well plates (Corning). After 24 h, the cells were treated with each drugs at the indicated concentrations and incubated for an additional 72 h. The viable cells were measured using Cell Counting Kit-8 solution (Dojindo, Rockville, MD, USA). Absorbance was measured at 450 nm after 3 h. Data were expressed as percent growth relative to untreated control cells.

### 2.9. Western Blotting and Antibodies

The cells were lysed in RIPA buffer supplement with leupeptin (0.5 μg/mL), aprotin (0.5 μg/mL), PMSF (1 mM), sodium orthovanadate (0.2 mM), and BGP (0.2 mM). Western blotting was then performed. Antibodies against TNS4 and V5-tag were purchased from Invitrogen. phospho-EGFR (Y1092), ERK, phospho-ERK1/2 (T202/Y204), and β-actin were purchased from Cell Signaling Technology (CST, Danvers, MA, USA). Anti-EGFR antibodies were purchased from Bethyl (Montgomery, AL, USA).

### 2.10. RNAi Studies

pLKO.shRNA plasmids targeting TNS4 and GFP were purchased from Sigma (St. Louis, CA, USA) and produced using protocols from the RNAi Consortium (http://www.broadinstitue.org/rnai/trc). Cells were prepared a day before infection and then incubated for 24 h with dilute virus containing media with 8 μg/mL polybrene. The transfected cells were treated with puromycin for 1 week.

### 2.11. Quantitative Real-Time PCR Analysis

Total cellular RNA was prepared from the cells by using an RNeasy Mini Kit (Qiagen, Hilden, Germany). Total RNA was used to synthesize the PrimeScript RT-PCR Kit (Takara Bio Inc., Shiga, Japan). Quantitative PCRs were performed with the use of SYBR green PCR Master Mix (Toyobo, Osaka, Japan) and we used an ABI7300 real-time PCR system (Applied Biosystems). GAPDH was used as the internal standard for normalization. The primer sequences used are as follows; TNS4 primer set (forward: 5′-CACCATGAAGTTCGTGATG-3′; reverse: 5′-CGGTATGAAGAGCTGTCC-3′), GAPDH primer set (forward: 5′-GGTGTGAACCATGAGAAGTATGA-3′; reverse: 5′-GAGTCCTTCCACGATACCAAAG-3′) [30,31].

## 3. Results

### 3.1. Whole Transcriptome Analysis Identified a Subset of Genes That Are Significantly Modulated by Cetuximab Treatment in Xenograft Mouse Tumors Induced by KRAS^G12D^ Mutation-Bearing LS174T CRC Cells

**Figure 1:**
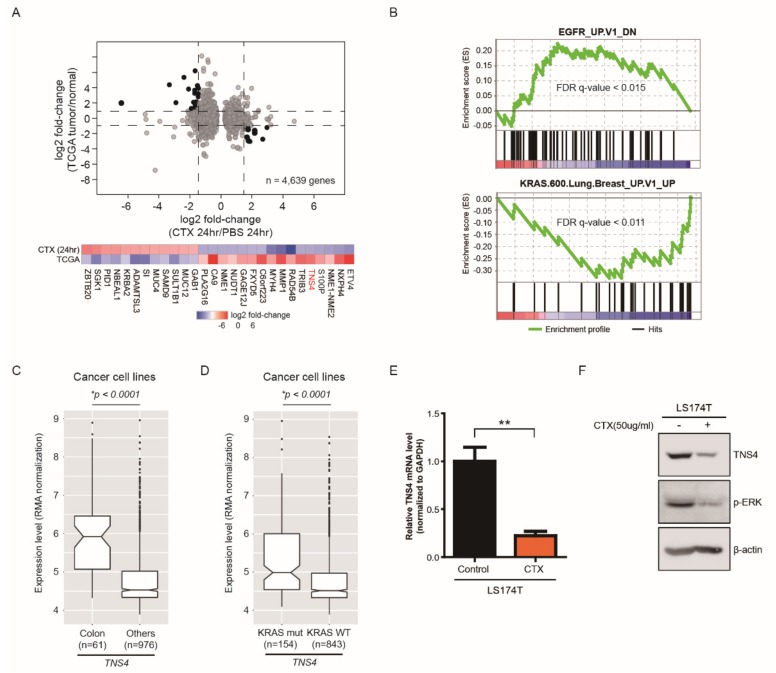
Identification of a subset of genes significantly regulated by cetuximab treatment through whole genome expression analysis. (**A**) Expression level of genes (FDR-adjusted *p*-value < 0.05) in cetuximab (CTX)-treated and PBS-treated xenograft mouse tumor with *KRAS*^G12D^ mutation-bearing LS174T cells were compared with the expression levels of genes in the COADREAD obtained from the cancer genome atlas (TCGA) database. The fold change of (CTX-treated/PBS-treated) or (tumor/normal) is shown in log2 in graph. (**B**) Gene set enrichment analysis (GSEA) for common biological function of EGFR or KRAS associated pathway in CTX treated LS174T cells is shown. (**C**,**D**) Box plots indicate the expression level of TNS4 in colon (n = 61) or other cancer cell lines (n = 976) (**C**) and in KRAS mutated (n = 154) or wild-type cell lines (n = 843) (**D**). *p*-value was calculated using the Mann-Whitney U test. (**E**) Quantitative PCR analysis was performed using primer sets targeting TNS4 on total mRNA isolated from LS174T cells treated with either PBS (control) or CTX. The bar graphs show the relative *TNS4* mRNA levels for CTX-treated samples were normalized to control (**, *p* < 0.01). (**F**) LS174T cells were incubated for 24 h with or without cetuximab and the resulting cell lysates were subjected to immunoblotting with antibodies against TNS4 and phospho-ERK. β-actin was used as loading control.

To identify a set of genes whose expression is regulated by cetuximab treatment in colorectal cancer cells harboring *KRAS* activating mutation, we first established xenograft mouse models with *KRAS*^G12D^ mutation-bearing LS174T cells, which was chosen as a representative colon cancer cell line for this study. Next, we carried out whole transcriptome analysis (mRNA-seq) in xenograft mouse tumors (n = 4 for each condition) obtained after 24 h of the subcutaneous injection of cetuximab or PBS (control). To avoid potential contamination from neighboring mouse tissues, we performed a series of bioinformatics analyses (see Methods). Correlation analysis for expression levels of the matched genes between the colorectal adenocarcinoma (COADREAD) tissues (tumor or adjacent-normal tissues) obtained from the cancer genome atlas (TCGA) database and LS174T cells revealed that the cell line well represented the COADREAD tumor transcriptome (R^2^ = 0.51), while the COADREAD adjacent-normal transcriptome showed less correlation with the cell line (R^2^ = 0.19) (Figure S1A,B). Using the RSEM and EBSeq analyses pipeline with an FDR cutoff of 0.05, we identified in total 1177 or 3462 differentially up- or down-regulated genes (DEGs), respectively, in cetuximab-treated tumors compared to PBS-treated tumors (Figure 1A). Then, we compared these DEGs to the expression levels of the matched genes in COADREAD tissues. By these integrated analyses, we finally selected a set of genes (n = 28) showing a substantially inversely correlated expression pattern between cetuximab-treated tumors and COADREAD patients (Figure 1A). We propose that these selected gene sets would serve as clinically significant potential biomarkers of cetuximab response because these genes are up-regulated (or down-regulated) in tumor tissues of CRC patients and conversely controlled by cetuximab treatment.

In addition, we conducted the gene set enrichment analysis (GSEA) with DEGs and confirmed that cetuximab treatment results in suppression of EGFR signaling pathways of LS174T cells (Figure 1B). Notably, we found that a subset of genes in KRAS signaling pathways is significantly modulated in LS174T cells by cetuximab, suggesting that cetuximab also exerts an effect on the oncogenic signaling pathways associated with mutant KRAS (Figure 1B). Further analysis of these selected genes with CCLE database identified that *TNS4*, a member of the Tensin protein family member involved in key cellular process including cell adhesion, migration and proliferation, was of great interest because it was specifically expressed in a colon cancer cell line (Figure 1C) as well as up-regulated in *KRAS* mutated cell lines compared to wild type cell lines (Figure 1D) [32,33]. In addition, notably, TNS4 expression was shown to be highly associated with overall survival of CRC patients [34]. Thus, we sought to pursue its functional significance in further studies.

### 3.2. TNS4 is Significantly Downregulated by Cetuximab Treatment in a Subset of CRC Cell Lines

**Figure 2:**
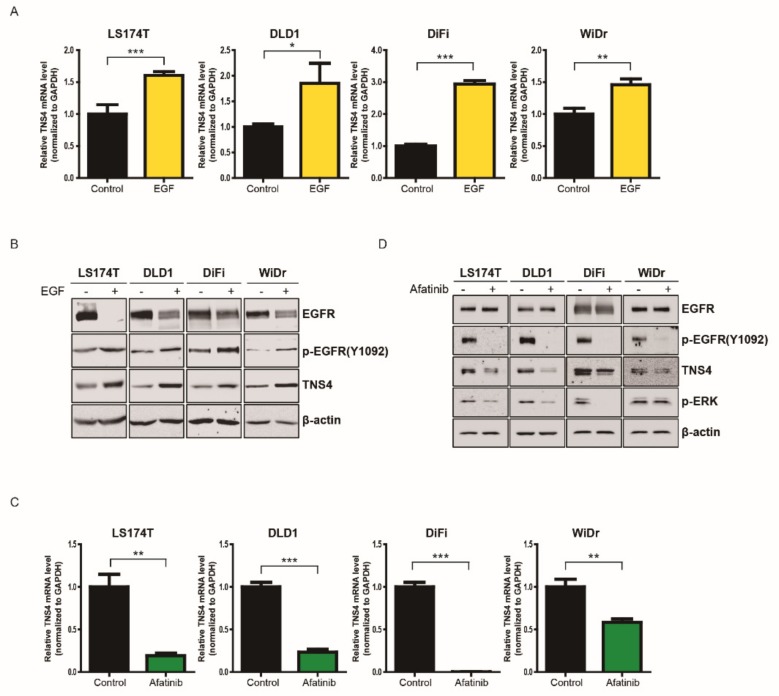
Expression of TNS4 in colon cancer cell lines are transcriptionally regulated via EGFR signaling pathways. (**A**,**C**) Quantitative PCR analysis was carried out using primer sets targeting *TNS4* on total mRNA isolated from LS174T, DLD1, DiFi and WiDr cells treated without (control) or with either EGF (10 ng/mL) for 12 h (**A**) or afatinib (1 μM) for 24 h. The bar graphs show the relative TNS4 mRNA levels of EGF-treated samples were normalized to control (*, *p* < 0.5; **, *p* < 0.01; and ***, *p* < 0.001). (**B**,**D**) Cell lysates prepared from LS174T, DLD1, DiFi and WiDr cells with or without EGF (**B**) or afatinib treatment (**D**) were subjected to immunoblotting analysis with antibodies against total EGFR or phospho-EGFR (Y1092), TNS4, phospho-ERK and β-actin.

First, to confirm cetuximab-induced downregulation of TNS4, we treated LS174T cells with cetuximab or PBS and examined both mRNA and protein levels of TNS4 by quantitative PCR (qPCR) with specific primers targeting TNS4 and immunoblotting analysis with anti-TNS4 antibody, respectively. Consistent with the data of mRNA profiling, both mRNA and protein levels of TNS4 were significantly decreased in the cells treated with cetuximab compared to PBS treatment, confirming that the expression of TNS4 in LS174T is downregulated by cetuximab (Figure 1E,F). Next, to explore whether cetuximab-induced downregulation of TNS4 also occurs in the other CRC cell lines, we repeated the same experiments with three available CRC cell lines harboring different genomic alterations, which includes DLD1 (*KRAS* G13D), DiFi (*EGFR* amplification) and WiDr (*BRAF* V600E) cells. It is known that DiFi cells are highly sensitive to cetuximab and the other cell lines show resistance to it because these cells harbor either *KRAS* or *BRAF* activating mutations [6,35,36]. Similar to the effect in LS174T cells, the expression of TNS4 in DLD1 and DiFi cells was diminished by cetuximab treatment in both mRNA and protein levels, compared to control cells (Figure S1C,D). Interestingly, the most significant change in *TNS4* mRNA levels was observed in DiFi cells, which is highly sensitive to this drug (Figure S1C). However, we did not observe any reduction of TNS4 expression in cetuximab-treated WiDr cells (Figure S1C,D). In addition, we found that levels of phospho-EGFR and phospho-ERK were reduced by cetuximab in LS174T, DLD1, and DiFi cells, but not in WiDr cells, which is correlated with TNS4 expression levels in these cells (Figure S1D). These results suggest that downregulation of TNS4 in LS174T, DLD1, and DiFi cells is caused by inhibition of activated EGFR signaling by cetuximab and that the lack of TNS4 reduction observed in WiDr is likely due to impaired suppression of EGFR signaling with cetuximab. To further validate the regulation of TNS4 by EGFR signaling pathways, we treated LS174T, DLD1, DiFi, and WiDr with EGF and examined the changes of *TNS4* mRNA and protein levels in these cells by RT-qPCR and immunoblotting analysis, respectively. In contrast to the inhibition of EGFR by cetuximab, EGFR activation with EGF increased the expression of TNS4 at mRNA and protein levels in all cell lines including WiDr (Figure 2A,B). Furthermore, afatinib, a dual kinase inhibitor of EGFR/ERBB2, led to significant downregulation of *TNS4* mRNA and protein levels in LS174T, DLD1, and DiFi cells, but modestly in WiDr cells (Figure 2C,D). Notably, similar to the results with cetuximab, while levels of phospho-ERK were robustly reduced by afatinib treatment in LS174T, DLD1 and DiFi cells, it was unaffected in WiDr cells under the same experimental condition. Taken together, we conclude that expression of TNS4 in LS174T, DLD1, and DiFi cell lines are transcriptionally regulated via EGFR signaling pathways.

### 3.3. TNS4 Expression Is Associated with Oncogenic Potential of Colon Cancer Cell Lines

**Figure 3:**
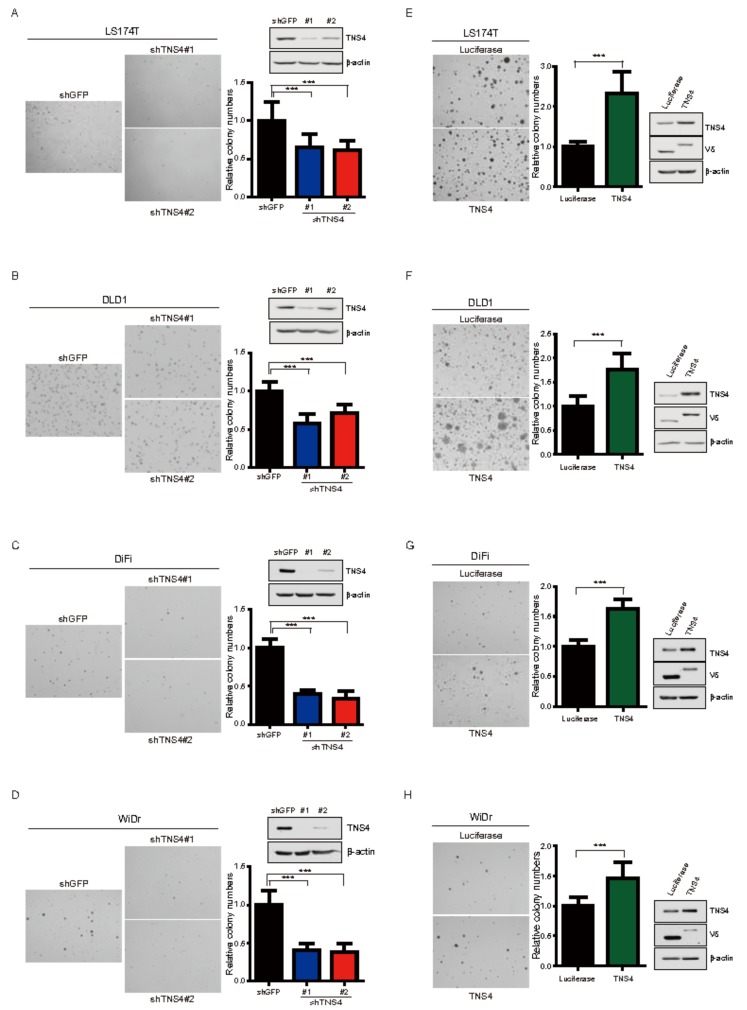
TNS4 expression is associated with the oncogenic potential of colon cancer cells. (**A**–**D**) LS174T (**A**), DLD1 (**B**), DiFi (**C**) and WiDr (**D**) cells stably expressing either shTNS4 or shGFP were used for colony formation assay in soft agar. The relative number of colonies formed by shTNS4 bearing cells are depicted as bar graphs (n = 3, mean + SD) following normalization to that of shGFP-bearing cells. Cell lysates prepared from the same cells were subjected to immunoblotting analysis with antibodies against TNS4. Representative photomicrographs of colonies formed in soft agar are shown. (**E**–**H**) LS174T (**E**), DLD1 (**F**), DiFi (**G**) and WiDr (**H**) cells ectopically expressing either TNS4 or luciferase were used for colony formation assay in soft agar. The bar graph depicts the relative number of colonies formed by TNS4 expressing cells following normalization to that of cells expressing luciferase (n = 3, mean + SD). Representative photomicrographs of colonies formed in soft agar are shown. Cell lysates prepared from LS174T, DLD1, DiFi and WiDr cells were subjected to immunoblotting with antibodies against TNS4 and V5-tag.

To investigate the effect of TNS4 on the oncogenic activity of colon cancer cells, we first introduced two plasmids encoding shRNA targeting the sequences of *TNS4* into LS174T, DLD1, DiFi, and WiDr cells along with a control shRNA targeting *GFP* by lentiviral infection and confirmed the specific ablation of TNS4 in the resulting cell lines by immunoblotting analysis. Compared to the control shGFP, the ability of anchorage-independent growth in soft agar was significantly reduced in all shTNS4 bearing cell lines (Figure 3A–D), demonstrating decreased TNS4 leading to the suppression of the oncogenic activity of LS174T, DLD1, DiFi, and WiDr cells. To further explore the oncogenic role of TNS4, we repeated the anchorage-independent growth assay with the established LS174T, DLD1, DiFi, and WiDr cells ectopically expressing either TNS4 or luciferase by introducing cognate cDNA via lentiviral transduction. The elevated expression of TNS4 in these cells were detected by immunoblotting. Compared to that of luciferase expressing cells, the number of colonies in soft agar was significantly increased in all TNS4 overexpressing cells (Figure 3E–H), showing that ectopic expression of TNS4 promotes the oncogenic growth of LS174T, DLD1, DiFi, and WiDr cells. Collectively, these results reveal that the levels of TNS4 expression in LS174T, DLD1, DiFi, and WiDr cells are closely correlated with their oncogenic growth, suggesting that TNS4 may play a crucial role in regulating the oncogenic potential of a subset of colorectal cancer cells.

### 3.4. TNS4 Expression Is Regulated by MAP Kinase Pathway at a Transcriptional Level

It is well known that aberrant activation of MAP kinase pathways induced by activating mutations in *KRAS* or *BRAF* contributes to abnormal cell growth and functions as a critical mechanism leading to tumorigenesis of colon cancer. Consistent with this notion, immunoblotting analysis revealed that phospho-ERK levels were constitutively elevated in LS174T, DLD1, and WiDr cells harboring *KRAS* or *BRAF* activating mutation. Interestingly, we observed that phospho-ERK was significantly lowered by cetuximab in LS174T and DLD1 cells (Figure S1D). The decreased levels of phospho-ERK in these cells were equivalent to that of DiFi cells treated with cetuximab (Figure S1D). Although it was expected that cetuximab would lead to suppression of ERK activation in DiFi cells harboring *EGFR* amplification, the finding that cetuximab was also able to inhibit the ERK activation in LS174T and DLD1 cells suggest that EGFR signaling pathways in part contribute to activation of ERK in the presence of *KRAS* mutation. However, given that these cell lines are refractory to cetuximab unlike in DiFi cells, the inhibition of ERK activation by cetuximab in LS174T and DLD1 cells may not be quantitatively and/or qualitatively sufficient in antitumor effect.

**Figure 4:**
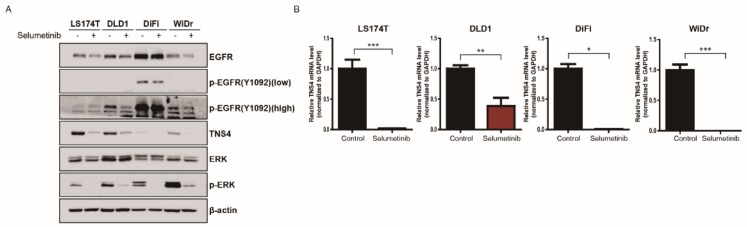
TNS4 expression is regulated by MAP kinase pathways at a transcriptional level. (**A**) LS174T, DLD1, DiFi and WiDr cells were incubated for 24 h with or without 1 μM selumetinib, and the resulting cell lysates were subjected to immunoblotting with the indicated antibodies. (**B**) The total mRNA isolated from LS174T, DLD1, DiFi, and WiDr cells treated with selumetinib (1 μM) for 24 h were used for qPCR analysis with primer sets targeting *TNS4*. The bar graphs show the relative mRNA levels of TNS4 in selumetinib-treated cells were normalized to that of untreated cells (*, *p* < 0.5; **, *p* < 0.01; and ***, *p* < 0.001).

Next, we sought to examine whether cetuximab-induced downregulation of TNS4 is mediated by MAP kinase signaling pathway. To this end, we treated LS174T, DLD1, DiFi, and WiDr cells with selumetinib, a specific inhibitor of MEK1/2 and determined the level of TNS4 in the resulting cells by immunoblotting. As expected, the treatment of selumetinib inhibited the level of phospho-ERK in all four cell lines (Figure 4A). Notably, we found that TNS4 expression was significantly decreased in LS174T, DLD1, DiFi, and WiDr cells treated with selumetinib, indicating that inhibition of MAP kinase pathways led to the downregulation of TNS4 (Figure 4A). Furthermore, we found that compared to control, TNS4 mRNA levels were significantly lowered in the cells treated with selumetinib by RT-qPCR analysis (Figure 4B), demonstrating that selumetinib-mediated downregulation of TNS4 may be caused at the transcriptional level. Similar observation was reported in a previous study [37].

### 3.5. Combinatorial Treatment with Selumetinib Increases the Sensitivity of Cetuximab in a Subset of KRAS Mutation-Bearing Colorectal Cancer Cell Lines

**Figure 5:**
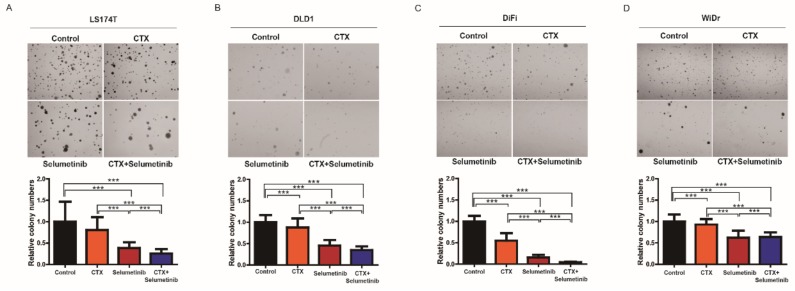
Combinatorial targeting of both EGFR and MAP kinase signaling pathways show synergistic effects against colorectal cancer cell lines. (**A**–**D**) Colony formation assay was performed with LS174T (**A**), DLD1 (**B**), DiFi (**C**) and WiDr (**D**) cells following treatment with either CTX (50 μg/mL or 10 μg/mL for DiFi) or selumetinib (0.1 μM) or together. The bar graph depicts the relative number of colonies formed by these cells following normalization to that of the same cells treated with PBS (control) (n = 3, mean + SD). Representative photomicrographs of colonies formed in soft agar are shown.

Given that the silencing of TNS4 has an impact on the oncogenic growth of LS174T, DLD1, DiFi, and WiDr cells and TNS4 expression is significantly downregulated by selumetinib, we next asked whether the cetuximab response is enhanced in these cells by combinatorial treatment with selumetinib. To this end, we assessed the ability of anchorage-independent growth of LS174T, DLD1, DiFi, and WiDr cells in soft agar in the presence of either cetuximab or selumetinib alone or both drugs in combination. Consistent with the previous reports, while the oncogenic growth of DiFi cells was significantly suppressed by cetuximab, LS174T, DLD1, and WiDr cells do not exert any effect on the growth under the same experimental condition [36]. In contrast, selumetinib treatment impeded the oncogenic growth of all four cell lines and these growth inhibitory effects were more profound when co-treated with cetuximab (Figure 5A–D). These results exhibit that combined inhibition of EGFR and aberrantly activated MAP kinase signaling pathway by drugs would be very effective against a subset of colon cancer cell lines including cetuximab-refractory *KRAS* or *BRAF* mutation-bearing cells. In order to further examine whether the observed growth inhibitory effects of cetuximab and selumetinib is mediated by downregulation of TNS4, we repeated the same experiments with the LS174T cells ectopically expressing either TNS4 or luciferase. Compared to control LS174T cells expressing luciferase, the number of colonies in soft agar formed by TNS4-expressing cells treated with either cetuximab, selumetinib or both drugs together were observed to be significantly higher under the same experimental condition, demonstrating that TNS4 overexpression led to decreased growth inhibitory effects by these drugs (Figure S2). These results suggest that TNS4 may serve as a key effector associated with cetuximab and selumetinib responses in LS174T cells.

## 4. Discussion

Despite much effort, no clinically applicable inhibitors targeting oncogenic KRAS mutants are yet available. As a result, there is currently no effective therapeutic strategy in treatment of CRC patients harboring such mutations. Traditionally, it is well accepted that the occurrence of activating mutations of *EGFR* and *KRAS* is mutually exclusive in tumors and EGFR-targeted therapy in the context of *KRAS* mutation is clinically ineffective [10,11,12,13]. Recently, two groups independently reported the interesting results that EGFR signaling pathways contribute to lung tumorigenesis driven by KRAS mutant, and consequently the inhibition of EGFR with MEK inhibitor promotes the antitumor effect in vivo and in vitro [22,23]. In the light of these findings, we sought to investigate whether this phenomenon holds true in CRC cancer driven by *KRAS* mutations with the following hypotheses. First, we postulated that some of genes whose expression is strongly modulated by cetuximab may play a crucial role in oncogenic potential of *KRAS* mutation-bearing CRC cancer. Second, effective targeting of their gene products alone or combination with inhibition of aberrantly activated MAP kinase signaling pathways may suppress the oncogenic activity of CRC tumors harboring *KRAS* or *BRAF* mutations.

By series of analyses, we identified twenty-eight genes whose expression are both significantly up- or downregulated in the xenograft mouse tumors driven by LS174T following cetuximab treatment and conversely expressed in CRC patients. We speculate that these selected genes are likely to be associated with the oncogenic potential of CRC cancer, as well as potential pharmacological targets of cetuximab. Considering the distinct tumor microenvironment between human and mouse, we cannot exclude the possibility that the selected genes may not have a complete physiological relevance. To avoid this issue, we focused on TNS4, a member of the Tensin protein family member, involved in a key cellular process including cell adhesion, migration, and proliferation, for further functional studies to test our hypotheses because TNS4 was found to be upregulated in various types of cancer and its expression is significantly correlated with *KRAS* mutation status in CRC cell lines based on our analysis [30,32,34,37,38,39,40].

Through functional analysis, we have confirmed that TNS4 is significantly downregulated by cetuximab in a subset of colorectal cancer cell lines including *KRAS* activating mutations, suggesting that TNS4 may be one of the pharmacological targets of cetuximab. In addition, we demonstrated that downregulation of TNS4 led to dramatic inhibition of the oncogenic growth of the CRC cell lines, implying that TNS4 plays a pivotal role as an oncoprotein in these cells. Importantly, this may provide the potential explanation to a recent report that high expression of TNS4 was associated with poor prognosis and distant metastasis in CRC patients [32,34,40,41]. Furthermore, we showed compelling evidence that TNS4 expression is transcriptionally regulated via EGFR and MAP kinase signaling pathway, which is consistent with others and our analysis that the higher TNS4 expression is observed in *KRAS* mutation-bearing CRC cell lines than in the other cell lines [32,37,42,43]. However, given that silencing of TNS4 in DiFi cells where the level of TNS4 appears to be relatively low still induced the comparable growth inhibitory effects shown in LS174T and DLD1 cells with high TNS4 expression, the oncogenic function of TNS4 may not be simply caused by increased levels of its expression. The detailed mechanism underlying how TNS4 contributes to oncogenic potential in CRC cell lines needs to be further investigated in future studies.

While the inhibition of the RAF-MEK-MAPK pathways with specific inhibitors showed the limited clinical activity for *KRAS*-mutation bearing cancers including CRC as a single agent, numerous preclinical and clinical studies have demonstrated that combinatorial approaches targeting multiple downstream effectors of KRAS exerted a more promising antitumor effect [44,45,46,47,48]. Notably, a recent report revealed that concomitant blockage of EGFR and MEK by combination treatment of pimasertib (MEK1/2 inhibitor) with cetuximab was proven to be effective against CRC cells harboring *KRAS* mutation [48]. Given that our results show that TNS4 appears to serve as a converged downstream effector of both EGFR and ERK signaling pathways, it is tempting to speculate that effective downregulation of TNS4 by the drugs targeting these signaling acts as a crucial determinant leading to antitumor effect in *KRAS* mutation-harboring CRC. In regard to this notion, it is intriguing to explore whether insufficient targeting of TNS4 is associated with resistance to current therapeutic approaches inhibiting RAF/MEK/MAPK pathways in CRC patients. In addition, several genes including MYC and β-catenin were known to be transcriptionally upregulated under the control of MAP kinase pathways [40,45,49]. Thus, it is possible that such genes may function synergistically with TNS4 to drive tumorigenesis, and as key effectors of drug responses. These possibilities remain to be further investigated in future studies. In summary, we showed that TNS4 is transcriptionally regulated by EGFR and MAPK pathways and plays a crucial role in the oncogenic potential of a subset of CRC cells harboring *KRAS* activating mutations. We propose that effective suppression of TNS4 by a single targeted agent or various combinatorial drugs may serve as a novel therapeutic strategy in treating a subset of cetuximab-refractory CRC patients harboring *KRAS* activating mutations.

## Supplementary Materials

The following are available online at https://www.mdpi.com/2073-4409/8/8/878/s1, Figure S1: TNS4 is significantly downregulated by cetuximab in a subset of colon cancer cell lines, Figure S2: TNS4 overexpression in LS174T cells decreased the efficacy of EGFR and MEK inhibitors.
