# Supplementary material for: Whole Transcriptome Analysis Identifies TNS4 as a Key Effector of Cetuximab and a Regulator of the Oncogenic Activity of KRAS Mutant Colorectal Cancer Cell Lines
Source: Cells. 2019 Aug 12;8(8):878. doi: 10.3390/cells8080878 (PMC6721647; doi:10.3390/cells8080878)
Supplement: Supplementary file 1 [file cells-08-00878-s001.pdf]

Supplemental Figures

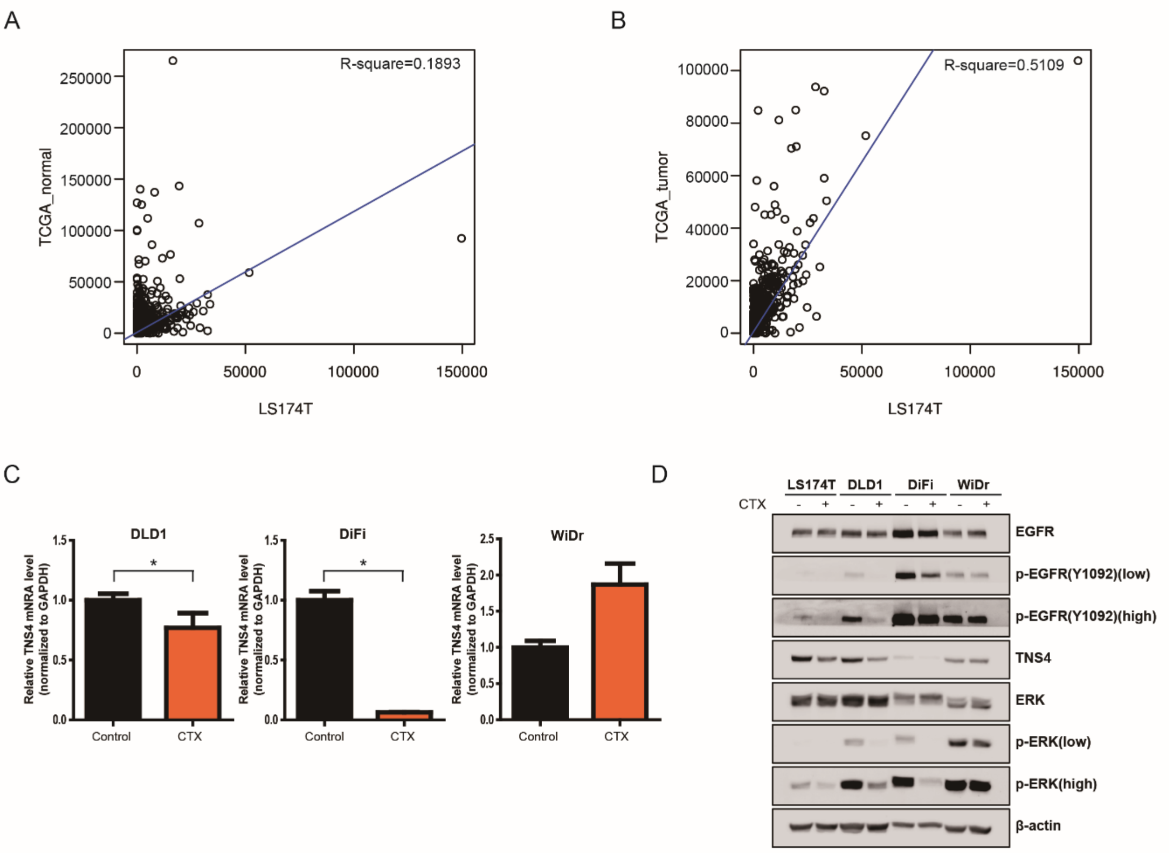

Figure S1. TNS4 is significantly downregulated by cetuximab in a subset of colon cancer cell lines. (**A–B**) Correlation analysis for expression levels of the matched genes between the COADREAD tissues (tumor or adjacent-normal tissues) obtained from the cancer genome atlas (TCGA) database and LS174T cells are shown. (**C**) The levels of *TNS4* mRNA in DLD1, DiFi and WiDr cells treated with and without cetuximab (50 μg/mL) for 24 hours were measured by quantitative PCR and represented as a graph following normalization to control (without CTX treatment). (D) LS174T, DLD1, DiFi and WiDr cells were incubated for 24 hours with or without 50 μg/mL CTX, and the resulting cell lysates were subjected to immunoblotting analysis with the indicated antibodies.

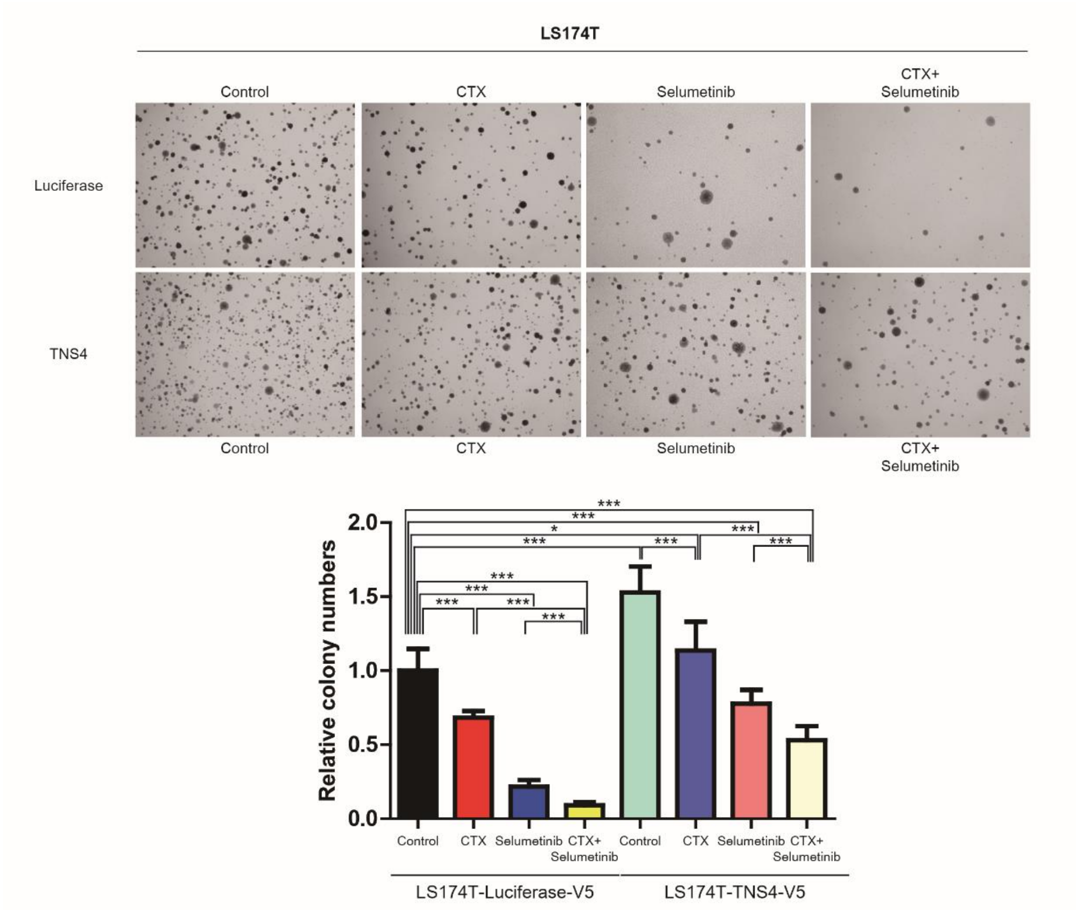

Figure S2. TNS4 overexpression in LS174T cells decreased the efficacy of EGFR and MEK inhibitors. LS174T cells stably expressing luciferase or TNS4 were used for colony formation in soft agar with either CTX (50 μg/mL) or selumetinib (0.1 μM) or together. The bar graph depicts the relative number of colonies formed by these cells following normalization to that of the same cells treated with PBS (control) (n = 3, mean + SD) (***, $P < 0.001$). Representative photomicrographs of colonies formed in soft agar are shown.
